# Effects of heart rate in modified look-locker inversion recovery (MOLLI) T1 mapping

**DOI:** 10.1186/1532-429X-15-S1-P135

**Published:** 2013-01-30

**Authors:** Yoshinori Suizuki, Kunihiko Teraoka, Yoshiaki Komori, Andreas Greiser

**Affiliations:** 1 Department of Radiology, Tokyo Medical university Hachoji Medical Center, Tokyo, Japan; 2 Department of cardiology, Tokyo Medical university Hachoji Medical Center, Tokyo, Japan; 3 Healthcare Sector, Siemens Japan K.K., Tokyo, Japan; 4 Healthcare Sector, Siemens AG, Erlangen, Germany

## Background

MOLLI is a method of producing a T1 map at end diastole by using the electrocardiogram (ECG) under respiratory arrest to obtain consecutive inversion recovery images with an arbitrary number of MOLLI cycles. Since a variety of heart rates are presumed clinically, it is important to understand their effects on the T1 value and the error of measurement. We investigated the error of measurement in MOLLI T1 mapping caused by changes in the heart rate.

## Methods

MAGNETOM Avanto 1.5T, simulation ECG, and a phantom with an aqueous dilution of Gd-DTPA were used. The phantom contents and T1 values were as follows: olive oil = 213 ms, physiological saline = 3122.9 ms and aqueous dilutions of Gd-DTPA 103.6 ms-1739.9 ms were used. The imaging conditions were as follows: Sequence is single-shot TrueFISP, TR = 700 ms, TE = 1.13 ms, FA = 35 deg, FOV = 360 mm, Resolution=256 x 65%, shot duration time = 123 ms, Bandwidth = 1028 Hz/Px, Reordering=linear, MOLLI TI start = 82 ms, MOLLI TI increment =18 ms resting heart cycle 0-8, iPAT (+). In the experiment, #1 the T1 value was measured by using RR = 800 ms and two sets of MOLLI cycles (LL1=4 images, LL2=4 images) and changing the number of resting heart cycles (RT) between LL1 and LL2 from 0 to 8, and #2 The T1 value was measured by varying RR in the range 400-1000 ms and both for the case of using two sets of MOLLI cycles (LL1=8 images, LL2=2 images) with RT fixed to be 1. and for the case of changing RT from 0 to 10 so that the IR interval between LL1 and LL2 becomes approximately 7200 ms.

## Results

**Figure 1:**
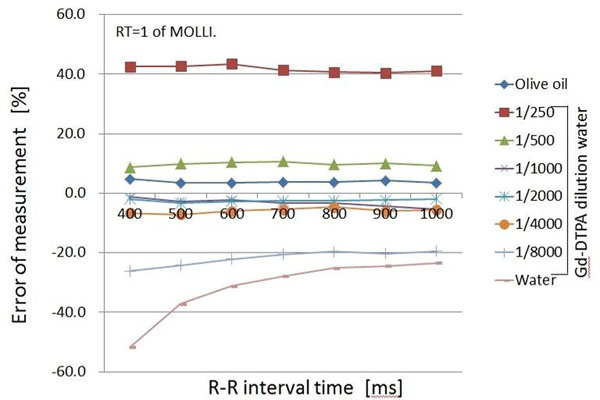
Comparison of R-R interval and error measurement by MOLLI (LL1 = 8 image, LL = 2 image, RT = 1)

**Figure 2:**
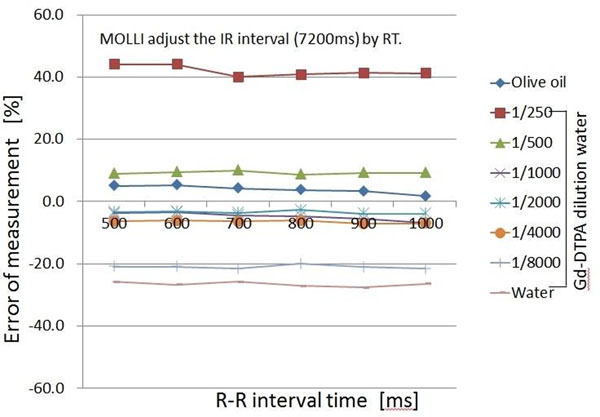
Comparison of R-R interval and error of measurement by MOLLI (LL1 = 8 image, LL = 2 image, RT = 0-7) adjust of RT (0-7) to make the IR interval constant 7200 ms

The results #1, The error of measurement was approximately 45% for a T1 value of 103.6ms and approximately 5% for T1 values of 347.8-1044.2 ms, with no dependence on RT. However, for T1 values in the range 1739.9-3122.9 ms, there were large changes with RT, in the range of 10-175%. The results #2, in which RT was fixed, With RT=1, as the heart rate increased the change in the error of measurement became greater, approximately 25-50% for T1 values of 1739.9-3122.9 ms. The results of varying RT are shown in Figure 1 and Figure 2. When RT was adjusted to make the IR interval constant the change in the error of measurement improved to approximately 20-27% for T1 values of 1739.9-3122.9 ms.

## Conclusions

When RT is fixed, the IR interval would vary depending on the heart rate. Especially in the case of high heart rates, it is possible that the error of measurement also changes more as the T1 value becomes longer (about 1000-3000 ms) due to the diminished signal strength when IR pulses are applied under conditions in which the recovery of the longitudinal magnetization is incomplete. Also, a factor which caused the error of measurement to become large for short T1 values (less than 200 ms) is the difficulty, with the duration time in this study, of capturing differences in the T1 value as image contrast because of the very fast recovery of the longitudinal magnetization, and the effects of the heart rate are considered to be small.

## Funding

Depending on the heart rate, there are greater effects on the change in the error of measurement for longer T1 values, and adjustment of RT to make the IR interval constant (longer than 7200ms) is effective as a method of stabilizing the error of measurement.

